# Transcriptome and translatome of CO_2_ fixing acetogens under heterotrophic and autotrophic conditions

**DOI:** 10.1038/s41597-021-00837-7

**Published:** 2021-02-09

**Authors:** Yoseb Song, Jiyun Bae, Jongoh Shin, Sangrak Jin, Jung-Kul Lee, Sun Chang Kim, Suhyung Cho, Byung-Kwan Cho

**Affiliations:** 1 Department of Biological Sciences, Korea Advanced Institute of Science and Technology, Daejeon, 34141 Republic of Korea; 2 Department of Chemical Engineering, Konkuk University, Seoul, 05029 Republic of Korea; 3 KAIST Institute for the BioCentury, Korea Advanced Institute of Science and Technology, Daejeon, 34141 Republic of Korea; 4 Intelligent Synthetic Biology Center, Daejeon, 34141 Republic of Korea

**Keywords:** Transcriptomics, Translation, Systems biology, Gene expression analysis

## Abstract

Acetogens are anaerobic bacteria that utilise gaseous feedstocks such as carbon monoxide (CO) and carbon dioxide (CO_2_) to synthesise biomass and various metabolites via the energetically efficient Wood-Ljungdahl pathway. Because of this pathway, acetogens have been considered as a novel platform to produce biochemicals from gaseous feedstocks, potentially replacing the conventional thermochemical processes. Despite their advantages, a lack of systematic understanding of the transcriptional and translational regulation in acetogens during autotrophic growth limits the rational strain design to produce the desired products. To overcome this problem, we presented RNA sequencing and ribosome profiling data of four acetogens cultivated under heterotrophic and autotrophic conditions, providing data on genome-scale transcriptional and translational responses of acetogens during CO_2_ fixation. These data facilitate the discovery of regulatory elements embedded in their genomes, which could be utilised to engineer strains to achieve better growth and productivity. We anticipate that these data will expand our understanding of the processes of CO_2_ fixation and will help in the designing of strains for the desired biochemical production.

## Abstract (summary)

**Table:** 

Measurement(s)	RNA • transcriptome • translatome
Technology Type(s)	RNA sequencing • ribosomal profiling by sequencing assay
Factor Type(s)	carbon source • growth conditions
Sample Characteristic - Organism	Acetobacterium woodii • Clostridium aceticum • Clostridium drakei • Eubacterium limosum
Sample Characteristic - Environment	anaerobic

Machine-accessible metadata file describing the reported data: 10.6084/m9.figshare.13625942

## Background & Summary

Over the past decades, the usage of fossil fuels, which is now responsible for 40% of carbon dioxide (CO_2_) emission^1,2^, has rapidly increased, causing damage to the environment through the generation of greenhouse gases. The demand for sustainable replacements for fossil fuels to obtain environmental and economic sustainability has, therefore, also been increasing. Microbial gas fermentation has recently drawn much attention as an alternative method to convert gaseous feedstocks (CO or CO_2_) into biofuels and biochemicals, owing to its low operation cost compared to the conventional thermochemical process^3–6^. Among the organisms capable of gas fermentation, acetogenic bacteria (acetogens) are considered one of the most promising potential platforms^7,8^.

A unique feature of acetogens is the presence of the Wood-Ljungdahl pathway (WLP) coding genes in their genomes^9^. Among the CO_2_ fixing pathways discovered to date, the WLP is the most energetically efficient pathway, investing only one molecule of ATP per acetate produced, whereas the other pathways are ATP sinking pathways^10–12^. The WLP reduces CO_2_ into formate using the electrons derived from the oxidation of molecular hydrogen, then to formyl-tetrahydrofolate (THF) using one molecule of ATP. Subsequently, methenyl-THF, methylene-THF, and methyl-THF are synthesised using two additional reduction powers, which vary according to the acetogen. Subsequently, the methyl group of methyl-THF is transferred to the CO dehydrogenase/acetyl-CoA synthase (CODH/ACS) complex to form acetyl-CoA, combining with the reduced CO and coenzyme A (CoA). The synthesised acetyl-CoA can be converted into acetate to recoup ATP or biomass building blocks.

In addition to the WLP, energy conservation systems play essential roles in acetogens by compensating for the energy required for biomass synthesis. These systems utilise the ferredoxin (Fd): NAD^+^ oxidoreductase (Rnf) and the Fd: H^+^ oxidoreductase (Ech) complexes to translocate ions across the membrane to create ion gradient^13,14^. The established gradient drives the ion back into the cell through the ATP synthase complex, generating the ATP needed for the cell. Along with the ATP synthesis system, electron bifurcation, which oxidises one electron donor and transfers electrons to two different electron acceptors, helps to overcome energetic barriers by reducing low reduction potential Fd via oxidisation of a relatively higher reduction potential hydrogen molecule, which can then reduce CO_2_ and the ion translocating complex^10,15,16^.

Along with the WLP and the energy conservation system, acetogens, similar to any other organism, contain intricate regulatory networks to control gene expression under different conditions. To date, a large number of acetogens have been sequenced to identify their genomic features; this further required a systematic understanding of their transcriptional and translational regulatory processes. In contrast to genomic studies, relatively few studies on acetogens’ transcriptomes and translatomes under autotrophic growth conditions have been published. The lack of uniformly generated RNA sequencing (RNA-Seq) and ribosome profiling (Ribo-Seq) data for acetogens has not only limited from obtaining knowledge on the cellular responses but also expanding potential genetic tools for strain engineering. RNA-Seq and Ribo-Seq can determine the strength of promoters and Shine-Dalgarno (SD) sequences in the 5´-untranslated regions, which regulate transcription and translation, respectively.

In this study, we determined changes in the transcriptional and translational responses of acetogens under autotrophic growth condition compared to heterotrophic growth conditions, using RNA-Seq and Ribo-Seq. RNA-Seq and Ribo-Seq were performed on four acetogen species, *Acetobacterium woodii*, *Clostridium aceticum*, *Clostridium drakei*, and *Eubacterium limosum* cultured under the two conditions. Although studies on *E. limosum* and a transcriptomic study on *C. drakei* have been described in previous studies by our group, this study provides a uniformly generated and processed dataset of the additional model acetogens, which allows the comparative analysis of the transcriptome and translatome of CO_2_ fixing acetogens^17,18^. The presented RNA-Seq and Ribo-Seq results will provide a fundamental understanding of the responses of the acetogens to autotrophic conditions, and thereby widen genetic tools for strain engineering to produce biochemicals using CO_2_ as a carbon building block.

## Methods

### Bacterial culture conditions

For this study, *A. woodii* DSM 1030, *C. aceticum* DSM 1496, *C. drakei* DSM 12750, and *E. limosum* DSM 20543 were obtained from the Leibniz Institute DSMZ-German Collection of Microorganisms and Cell Cultures (DSMZ, Braunschweig, Germany). *A. woodii*, *C. aceticum*, and *C. drakei* were cultured under strict anaerobic conditions at 30 °C and *E. limosum* was cultured under anaerobic conditions at 37 °C in 150 mL serum bottles filled with 100 mL DSMZ 135 medium (pH 7.0), which is composed of 1 g L^−1^ NH_4_Cl, 2 g L^−1^ yeast extract, 10 g L^−1^ NaHCO_3_, 0.1 g L^−1^ MgSO_4_ × 7H_2_O, 0.3 g L^−1^ cysteine-HCl, 10 mL vitamin solution (4 mg L^−1^ biotin, 4 mg L^−1^ folic acid, 20 mg L^−1^ pyridoxine-HCl, 10 mg L^−1^ Thiamine-HCl, 10 mg L^−1^ riboflavin, 10 mg L^−1^ nicotinic acid, 10 mg L^−1^ pantothenate, 0.2 mg L^−1^ Vitamin B_12_, 10 mg L^−1^ p-aminobenzoic acid and 10 mg L^−1^ lipoic acid), 20 mL trace element solution (1.0 g L^−1^ Nitrilotriacetic acid, 3.0 g L^−1^ MgSO_4_ × 7H_2_O, 0.5 g L^−1^ MnSO_4_ × H_2_O, 1.0 g L^−1^ NaCl, 0.1 g L^−1^ FeSO_4_ × 7H_2_O, 180 mg L^−1^ CoSO_4_ × 7H_2_O, 0.1 g L^−1^ CaCl_2_ × 2H_2_O, 180 mg L^−1^ ZnSO_4_ × 7H_2_O, 10 mg L^−1^ CuSO_4_ × 5H_2_O, 20 mg L^−1^ KAI(SO_4_)_2_ × 12H_2_O, 10 mg L^−1^ H_3_BO_3_, 10 mg L^−1^ Na_2_MO_4_ × 2H_2_O, 30 mg L^−1^ NiCl_2_ × 6H_2_O, 0.3 mg L^−1^ Na_2_SeO_3_ × 5 H_2_O, 0.4 mg L^−1^ Na_2_WO_4_ × 2H_2_O), 4.6 mM KH_2_PO_4_, 5.4 mM K_2_HPO_4_, and 4 µM resazurin. For heterotrophic growth, *A. woodii*, *C. aceticum*, and *C. drakei* were cultivated in the fructose supplemented (5 g L^−1^) media and *E. limosum* was cultured in glucose supplemented (5 g L^−1^) media. For autotrophic growth, all of the strains were cultivated in DSMZ 135 media containing H_2_/CO_2_ (80:20) with pressure of 200 kPa in the headspace (50 mL). The media used for culturing *A. woodii* was supplemented with 2 g L^−1^ NaCl. For the main culture, the precultured cells were harvested via anaerobic centrifugation, then washed with basal DSMZ 135 media three times and inoculated in 100 mL fresh DSMZ 135 media supplemented with corresponding carbons. All of the strains were cultured in biological duplicates.

### RNA-Seq library preparation

Duplicate samples were harvested at the mid-exponential phase by centrifugation at 4,000 *g* for 15 min at 4 °C. The collected cells were resuspended anaerobically in 500 µL of lysis buffer, comprising 20 mM Tris-HCl (pH 7.4), 140 mM NaCl, 5 mM MgCl_2_, and 1% Triton X-100. Liquid nitrogen was used to freeze the samples, which were then ground using a mortar and pestle. Lysed cells were thawed on ice, and the debris was removed by centrifugation at 4,000 *g* for 15 min at 4 °C. Subsequently, the total RNA was isolated using TRIzol (Thermo Scientific, Waltham, MA, USA) according to the manufacturer’s instruction. To remove the remaining genomic DNA (gDNA), the RNA was treated with 4 U of rDNase I (Ambion, Austin, TX, USA) for 1 h at 37 °C, then incubated at 75 °C for 10 min to deactivate the enzyme. To remove ribosomal RNAs (rRNA) in the gDNA-depleted RNA, the Ribo-Zero^TM^ rRNA Removal Kit for Meta-bacteria (Epicentre, Madison, WI, USA) was used according to the manufacturer’s instruction. The quality of the rRNA-depleted RNA was checked using an Agilent 2200 TapeStation system (Agilent Technologies, Santa Clara, CA, USA). To construct the libraries for RNA-seq, the TrueSeq Stranded mRNA Library Prep Kit (Illumina, San Diego, CA, USA) was used on the quality confirmed RNA. The libraries were sequenced using the 150 bp read recipe with an Illumina MiSeq^TM^ system.

### Ribo-Seq library preparation

For Ribo-Seq, 100 µM chloramphenicol (CM) was added to the cultures which were then further incubated at 30 °C or 37 °C, corresponding to the duplicate culture conditions, for 10 min. The CM treated cells were subsequently washed using 500 µL polysome buffer composed of 20 mM Tris-HCl (pH 7.4), 140 mM NaCl, 5 mM MgCl_2_, and 100 µM CM, and resuspended in lysis buffer consisting of 20 mM Tris-HCl (pH 7.4), 140 mM NaCl, 5 mM MgCl_2_, 100 µM CM, and 1% Triton X-100. The resuspended cells were frozen in liquid nitrogen and ground with a pestle and mortar. The powdered cells were recovered by centrifugation at 4,000 *g* for 15 min at 4 °C, and the resultant supernatant was additionally centrifuged at 16,000 *g* for 10 min at 4 °C. To degrade RNA unprotected by ribosomes, 400 U MNase (NEB, Ipswich, MA, USA), 2 µL bovine serum albumin (1 mg mL^−1^), and 20 µL of 10 × MNase buffer were added and samples were incubated at 37 °C for 2 h with gentle rotation. To inactivate the reaction, 10 µL 0.5 M EGTA (Sigma-Aldrich, St. Louis, MO, USA) was added to the sample. The monosome fraction was recovered using Microspin S-400 HR columns (GE Healthcare Life Sciences, Marlborough, MA, USA). The recovered ribosome-bound RNA was isolated using TRIzol, and the remaining rRNAs were removed with the Ribo-Zero^TM^ rRNA Removal Kit for Meta-bacteria. For the phosphorylation reaction, samples were denatured at 80 °C for 90 s, equilibrated to 37 °C, and incubated at 37 °C for 1 h with 5 µL 10 × T4 PNK buffer (NEB), 20 U SUPERase-In RNase Inhibitor, and 10 U T4 PNK (NEB). After purification of the RNA samples using RNeasy MinElute Column (Qiagen, Hilden, Germany), the concentration of purified RNA was measured using the Qubit RNA HS assay kit (Invitrogen, Carlsbad, CA, USA). For library construction, the small RNA library prep kit for Illumina (NEB) was used, and the constructed library was sequenced using the 50 bp read recipe on an Illumina Hiseq2500.

### Data processing

For RNA-Seq, the adapter sequence of the sequenced reads and quality below Phred score of 20 were trimmed. Trimmed reads shorter than 20 bp were discarded to improve the accuracy of the mapping result. Using CLC Genomics Workbench (CLC Bio, Aarhus, Denmark), the trimmed reads were mapped onto the *A. woodii* (NC_016894), *C. aceticum* (NZ_CP009687), *C. drakei* (NZ_CP020953), and *E. limosum* (NZ_CP019962) genomes using default parameters (mismatch cost = 2, deletion cost = 3, insertion cost = 3, length fraction = 0.9, and similarity fraction = 0.9) and only the uniquely mapped reads were rescued. The gene expression was calculated from the mapped read count statistics using the DESeq. 2 package in R with default parameters. For Ribo-Seq, the adaptors of the generated reads and quality below Phred score of 20 were removed, then trimmed with the same parameters applied for RNA-Seq, and reads shorter than 20 bp were again removed. The reads were mapped onto the *A. woodii* (NC_016894), *C. aceticum* (NZ_CP009687), *C. drakei* (NZ_CP020953), and *E. limosum* (NZ_CP019962) genomes using the default parameters (mismatch cost = 2, deletion cost = 3, insertion cost = 3, length fraction = 0.9, and similarity fraction = 0.9) and only the uniquely mapped reads were rescued. Using the obtained reads, the gene expression was normalised using the DESeq. 2 package in R with default parameters.

## Data Records

The RNA-Seq and Ribo-Seq datasets for *A. woodii* were deposited in the European Nucleotide Archive under study accession PRJEB33460^19^. The RNA-Seq and Ribo-Seq datasets for *C. aceticum* were deposited in the European Nucleotide Archive under study accession PRJEB36134^20^. The RNA-Seq and Ribo-Seq datasets for *C. drakei*, published in previous study^18^, were deposited in the NCBI Gene Expression Omnibus (GEO) repository with the accession code GSE118519 and the European Nucleotide Archive under study accession PRJEB36135^21,22^. The RNA-Seq and Ribo-Seq datasets for *E. limosum*, published in previous study, were deposited in the NCBI Gene Expression Omnibus (GEO) repository with the accession code GSE97613^17,23^. Detailed information on analysis of RNA-Seq and Ribo-Seq has been deposited in Figshare^24^.

## Technical Validation

**Fig. 1:**
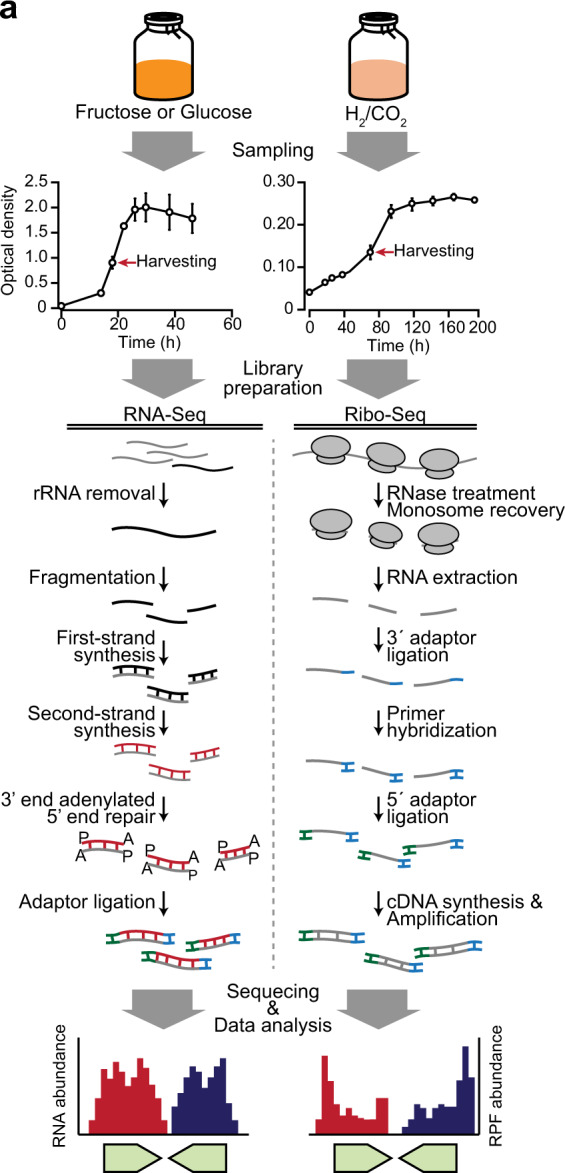
Overall experimental scheme of RNA sequencing (RNA-Seq) and ribosome profiling (Ribo-Seq) of acetogens. General strategy for library construction; under the heterotrophic (fructose or glucose supplemented media) and autotrophic growth conditions, cells were harvested at the mid-exponential phase, and construction of libraries for RNA-Seq and Ribo-Seq was carried out.

Acetogens have drawn much attention due to their ability to fix CO_2_ using the efficient WLP and the energy conservation system. To systematically understand this metabolism, four acetogen species, *A*. *woodii*, *C*. *aceticum*, *C*. *drakei*, and *E*. *limosum* were cultivated under heterotrophic or autotrophic condition, then sampled at the corresponding mid-exponential point (Fig. 1). RNA-Seq and Ribo-Seq libraries were created and sequenced using the Illumina platforms. For RNA-Seq, total bases of 1,989,686,915 nt, 3,309,840,659 nt, 3,061,597,892 nt, and 4,122,893,029 nt for *A. woodii*, *C. aceticum*, *C. drakei*, and *E. limosum*, respectively, were generated as raw data (Table 1). After obtaining the raw data, the adaptor sequences and poor-quality reads of lower than 99.9% accuracy were removed, resulting in 1,925,462,358 nt, 3,192,358,774 nt, 2,898,139,861 nt, and 4,033,415,284 nt, for *A. woodii*, *C. aceticum*, *C. drakei*, and *E. limosum*, with average read lengths of 147.8 nt, 132.8 nt, 141.0 nt, and 137.7 nt, respectively (Table 1). For Ribo-Seq, 30,075,896,511 nt, 15,893,868,174 nt, 26,109,101,619 nt, and 36,178,949,211 nt were produced in total as raw data for *A. woodii*, *C. aceticum*, *C. drakei*, and *E. limosum*, respectively (Table 2). The reads were then trimmed to remove the adaptor sequence and low-quality sequences, resulting 10,937,323,767 nt, 5,821,740,643 nt, 13,645,439,108 nt, and 5,132,234,720 nt total, for *A. woodii*, *C. aceticum*, *C. drakei*, and *E. limosum*, with average read lengths of 26.6 nt, 34.6 nt, 32.3 nt, and 36.4 nt, respectively (Table 2). The average length of the trimmed reads for RNA-Seq corresponds to the targeted sequencing length of 150 nt. In contrast, the lengths of the filtered Ribo-Seq reads ranged between 26 nt and 36 nt, which is shorter than the intended length of 50 bp. However, the length of the Ribo-Seq reads correspond to the actual lengths of mRNA protected by the ribosome during translation (between 20 to 40 nt)^25,26^.

**Table 1:** Summary statistics of RNA-Seq data of four acetogens.

Condition			H1	H2	A1	A2
***A. woodii***	**Raw**	Total Reads	1,411,725	1,788,006	5,800,707	4,443,182
		Avg. length (nt)	142.7	142.9	150.0	149.2
		Total Bases	201,512,022	255,472,170	869,919,174	662,783,549
	**Filtered Data**	Discarded Reads	62,776	91,493	144,789	117,181
		Filtered Reads	1,348,949	1,696,513	5,655,918	4,326,001
		Avg. length (nt)	142.6	142.2	149.8	149.1
		Filtered Bases	191,877,329	241,284,543	847,490,486	644,810,000
	**Mapped Data**	Mapped Reads	1,216,393	1,534,261	4,708,159	3,750,712
		Avg. length (nt)	142.4	142.4	149.9	149.1
		Mapped Bases	173,191,739	218,539,243	705,704,589	559,149,623
		% Mapped Bases	90.26	90.57	83.27	86.72
		Coverage (X)	42.8	54.0	174.5	138.2
***C. aceticum***	**Raw**	Total Reads	8,592,897	6,693,980	4,668,320	4,818,249
		Avg. length (nt)	127.3	121.9	146.8	148.3
		Total Bases	1,093,780,182	816,081,071	685,528,790	714,450,616
	**Filtered Data**	Discarded Reads	235,413	204,775	133,342	156,426
		Filtered Reads	8,357,484	6,489,205	4,534,978	4,661,823
		Avg. length (nt)	126.3	120.4	146.6	148.1
		Filtered Bases	1,055,763,278	781,447,377	664,855,961	690,292,158
	**Mapped Data**	Mapped Reads	4,329,443	2,632,789	4,387,537	4,477,253
		Avg. length (nt)	125.2	125.1	146.7	148.1
		Mapped Bases	541,809,667	329,356,872	643,597,092	663,159,147
		% Mapped Bases	51.32	42.15	96.80	96.07
		Coverage (X)	129.0	78.4	153.2	157.8
***C. drakei***	**Raw**	Total Reads	7,591,648	6,717,914	4,479,613	2,834,961
		Avg. length (nt)	141.6	142.5	141.3	139.8
		Total Bases	1,075,191,613	957,090,225	633,010,369	396,305,685
	**Filtered Data**	Discarded Reads	347,481	309,807	215,832	190,084
		Filtered Reads	7,244,167	6,408,107	4,263,781	2,644,877
		Avg. length (nt)	141.1	141.9	140.7	138.6
		Filtered Bases	1,022,112,572	909,413,672	600,048,804	366,564,813
	**Mapped Data**	Mapped Reads	6,995,218	6,177,415	4,108,521	2,224,333
		Avg. length (nt)	141.1	142.0	140.7	138.9
		Mapped Bases	987,123,830	877,112,720	578,089,707	308,847,185
		% Mapped Bases	96.58	96.45	96.34	84.25
		Coverage (X)	173.3	154.0	101.5	54.2
***E. limosum***	**Raw**	Total Reads	8,661,745	5,684,011	7,913,015	7,622,057
		Avg. length (nt)	138.4	138.1	136.0	139.5
		Total Bases	1,198,940,239	785,064,444	1,075,957,753	1,062,930,593
	**Filtered Data**	Discarded Reads	183,777	134,884	125,279	144,975
		Filtered Reads	8,477,968	5,549,127	7,787,736	7,477,082
		Avg. length (nt)	138.1	137.8	135.7	139.2
		Filtered Bases	1,170,988,282	764,581,763	1,056,867,399	1,040,977,840
	**Mapped Data**	Mapped Reads	8,322,146	5,426,527	7,316,337	6,141,327
		Avg. length (nt)	138.1	137.8	135.6	138.7
		Mapped Bases	1,149,126,214	747,505,718	991,993,490	851,487,524
		% Mapped Bases	98.13	97.77	93.86	81.80
		Coverage (X)	259.8	169.0	224.3	192.5

**Table 2:** Summary statistics of Ribo-Seq data of four acetogens.

Condition			H1	H2	A1	A2
***A. woodii***	**Raw**	Total Reads	249,172,796	69,476,513	131,703,279	139,370,873
		Avg. length (nt)	51.0	51.0	51.0	51.0
		Total Bases	12,707,812,596	3,543,302,163	6,716,867,229	7,107,914,523
	**Filtered Data**	Discarded Reads	76,432,862	23,198,952	37,668,910	41,268,313
		Filtered Reads	172,748,934	46,277,561	94,034,369	98,102,560
		Avg. length (nt)	35.0	33.7	35.4	36.3
		Filtered Bases	6,044,585,328	1,559,886,879	3,332,851,560	3,563,763,432
	**Mapped Data**	Mapped Reads	57,658,183	16,387,467	22,207,525	26,810,969
		Avg. length (nt)	34.4	33.9	36.0	37.4
		Mapped Bases	1,985,505,635	554,865,190	799,430,281	1,002,976,255
		% Mapped Bases	32.85	35.57	23.99	28.14
		Coverage (X)	490.9	137.2	197.6	248.0
***C. aceticum***	**Raw**	Total Reads	67,168,834	74,581,474	66,485,438	103,408,728
		Avg. length (nt)	51.0	51.0	51.0	51.0
		Total Bases	3,425,610,534	3,803,655,174	3,390,757,338	5,273,845,128
	**Filtered Data**	Discarded Reads	33,400,566	36,063,360	28,299,311	45,665,696
		Filtered Reads	33,768,268	38,518,114	38,186,127	57,743,032
		Avg. length (nt)	33.2	35.8	34.5	34.7
		Filtered Bases	1,122,582,742	1,377,659,778	1,318,798,272	2,002,699,851
	**Mapped Data**	Mapped Reads	10,186,053	13,323,148	2,460,004	4,919,132
		Avg. length (nt)	32.2	37.1	33.0	34.2
		Mapped Bases	328,229,231	493,590,577	81,089,933	168,361,829
		% Mapped Bases	29.24	35.83	6.15	8.41
		Coverage (X)	78.1	117.5	19.3	40.1
***C. drakei***	**Raw**	Total Reads	128,106,986	151,475,714	104,518,388	127,842,081
		Avg. length (nt)	51.0	51.0	51.0	51.0
		Total Bases	6,533,456,286	7,725,261,414	5,330,437,788	6,519,946,131
	**Filtered Data**	Discarded Reads	24,282,933	24,402,980	19,528,167	21,382,100
		Filtered Reads	103,824,053	127,072,734	84,990,221	106,459,981
		Avg. length (nt)	33.2	32.7	32.1	31.2
		Filtered Bases	3,442,391,045	4,151,411,664	2,725,965,604	3,325,670,795
	**Mapped Data**	Mapped Reads	6,691,311	16,666,137	1,995,225	2,786,678
		Avg. length (nt)	35.9	35.6	35.4	35.7
		Mapped Bases	240,455,393	593,484,191	70,552,856	99,558,665
		% Mapped Bases	6.99	14.30	2.59	2.99
		Coverage (X)	42.2	104.2	12.4	17.5
***E. limosum***	**Raw**	Total Reads	161,051,276	194,196,692	176,529,086	177,614,107
		Avg. length (nt)	51.0	51.0	51.0	51.0
		Total Bases	8,213,615,076	9,904,031,292	9,002,983,386	9,058,319,457
	**Filtered Data**	Discarded Reads	117,782,216	148,833,839	151,908,684	149,812,894
		Filtered Reads	43,269,060	45,362,853	24,620,402	27,801,213
		Avg. length (nt)	38.0	35.1	36.7	35.8
		Filtered Bases	1,644,643,961	1,591,099,387	902,716,987	993,774,385
	**Mapped Data**	Mapped Reads	22,576,465	29,673,717	14,399,376	17,294,237
		Avg. length (nt)	33.3	32.2	34.5	33.5
		Mapped Bases	751,347,321	956,182,329	496,810,251	580,030,984
		% Mapped Bases	45.68	60.10	55.03	58.37
		Coverage (X)	169.9	216.2	112.3	131.1

**Fig. 2:**
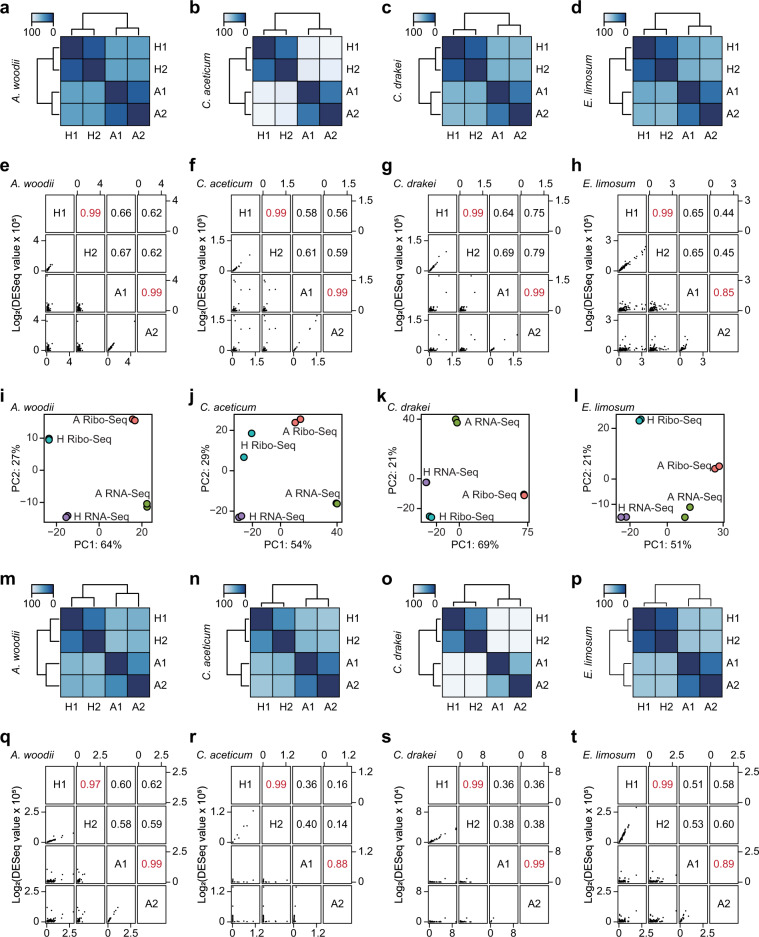
Quality of RNA sequencing (RNA-Seq) and ribosome profiling (Ribo-Seq) data. Reproducibility of RNA-Seq of *A. woodii* (**a**), *C. aceticum* (**b**), *C. drakei* (**c**), and *E. limosum* (**d**) cultivated under heterotrophic (H1 and H2) and autotrophic (A1 and A2) conditions. Pairwise correlation of RNA-Seq of *A. woodii* (**e**), *C. aceticum* (**f**), *C. drakei* (**g**), and *E. limosum* (**h**) cultivated under heterotrophic (H1 and H2) and autotrophic (A1 and A2) conditions. Principal components analysis of RNA-Seq and Ribo-Seq of *A. woodii* (**i**), *C. aceticum* (**j**), *C. drakei* (**k**), and *E. limosum* (**l**) cultivated under the heterotrophic (H: coloured purple and blue) and autotrophic (A: coloured green and red) conditions for RNA-Seq and Ribo-Seq. Reproducibility of Ribo-seq of *A. woodii* (**m**), *C. aceticum* (**n**), *C. drakei* (**o**), and *E. limosum* (**p**) cultivated under heterotrophic (H1 and H2) and autotrophic (A1 and A2) conditions. Pairwise correlation of Ribo-Seq of *A. woodii* (**q**), *C. aceticum* (**r**), *C. drakei* (**s**), and *E. limosum* (**t**) cultivated under heterotrophic (H1 and H2) and autotrophic (A1 and A2) conditions.

**Fig. 3:**
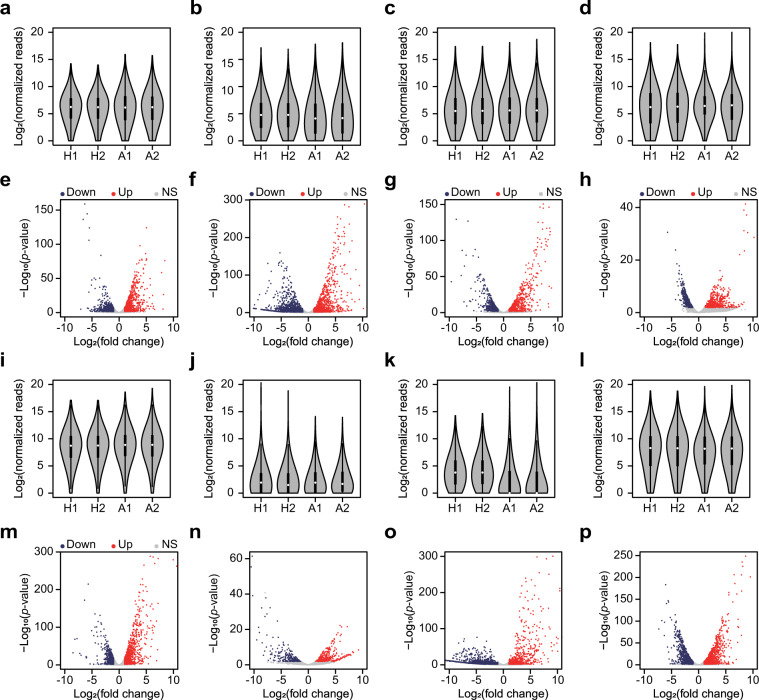
Normalized distribution of RNA sequencing (RNA-Seq) and ribosome profiling (Ribo-Seq) data. Distribution of normalised reads of RNA-Seq under the heterotrophic (H1 and H2) and the autotrophic (A1 and A2) conditions of *A. woodii* (**a**), *C. aceticum* (**b**), *C. drakei* (**c**), and *E. limosum* (**d**). Volcano plots of RNA-Seq of *A. woodii* (**e**), *C. aceticum* (**f**), *C. drakei* (**g**), and *E. limosum* (**h**) under the heterotrophic and the autotrophic conditions, with blue and red dots indicating significantly down and upregulated genes, respectively. Distribution of normalised reads of Ribo-Seq result under heterotrophic (H1 and H2) and autotrophic (A1 and A2) conditions of *A. woodii* (**i**), *C. aceticum* (**j**), *C. drakei* (**k**), and *E. limosum* (**l**). Volcano plots of *A. woodii* (**m**), *C. aceticum* (**n**), *C. drakei* (**o**), and *E. limosum* (**p**) under heterotrophic and autotrophic conditions, with blue and red dots indicating significantly down and upregulated genes, respectively.

Following the quality control process, the filtered reads were mapped to the corresponding genome sequences (see Method section for detail). For RNA-Seq, the total number of bases mapped to *A. woodii*, *C. aceticum*, *C. drakei*, and *E. limosum* were 1,656,585,194 nt, 2,177,922,778 nt, 2,751,173,442 nt, and 3,740,112,946 nt, respectively, with a minimum coverage value of 42.8 folds, which is sufficient to determine differential gene expression (Table 1). The mapped RNA-Seq reads were normalised using the DESeq. 2 package in R to determine reproducibility of the samples under the target conditions using hierarchical clustering (Fig. 2a–h) and principal component analysis (Fig. 2i–l), which validates that the biological replicates were reproducible within the same growth conditions. The range of the mapped normalised reads of the acetogens under the conditions were consistent (Fig. 3a–d). Subsequently, to identify differentially expressed genes (DEGs), fold changes between the heterotrophic and the autotrophic conditions were calculated with their associated *P*-values. Genes with fold changes of >2 or <0.5, respectively, and *P*-value < 0.01 were defined as up- and down-regulated DEGs, respectively. A total of 685, 793, 600, and 406 upregulated DEGs, and 500, 940, 582, and 505 downregulated DEGs under autotrophic conditions were identified by RNA-seq for *A. woodii*, *C. aceticum*, *C. drakei*, and *E. limosum*, respectively (Fig. 3e–h).

Similar to the RNA-Seq data, the Ribo-Seq reads were normalised using DESeq. 2, and the reproducibility of the replicates was confirmed via hierarchical clustering (Fig. 2m–t). Following this, principal component analysis validated that the Ribo-Seq expression patterns of the replicates were highly correlated only within the conditions (Fig. 2i–l). In addition, the range of the normalised Ribo-Seq reads were consistent in all acetogens (Fig. 3i–l). The verified reads were used to determine translational DEGs of the four acetogens under autotrophic conditions. This identified 745, 280, 501, and 854 significantly upregulated genes and 478, 196, 1,417, and 868 significantly downregulated genes from *A. woodii*, *C. aceticum*, *C. drakei*, and *E. limosum*, respectively (Fig. 3m–p).

## Data Availability

The version and parameter of all bioinformatics tools used in this work are described in the Methods section.
